# Assessing the ability of white-rot fungi to tolerate polychlorinated biphenyls using predictive mycology

**DOI:** 10.1080/21501203.2018.1481152

**Published:** 2018-06-08

**Authors:** Marcela Alejandra Sadañoski, Juan Ernesto Velázquez, María Isabel Fonseca, Pedro Darío Zapata, Laura Noemí Levin, Laura Lidia Villalba

**Affiliations:** a Laboratorio de Biotecnología Molecular, Instituto de Biotecnología Misiones, CONICET, Facultad de Ciencias Exactas Químicas y Naturales, Universidad Nacional de Misiones, Posadas, Argentina; b Laboratorio de Micología Experimental, Dpto. de Biodiversidad y Biología Experimental, FCEN, UBA, INMIBO (CONICET), CABA, Argentina

**Keywords:** Polychlorinated biphenyls, white-rot fungi, predictive mycology, modelling, laccase

## Abstract

The aim of the present study was to assess the ability of different white-rot fungi to tolerate polychlorinated biphenyls (PCBs) using predictive mycology, by relating fungal growth inhibition to ligninolityc enzyme secretion. Fungal strains were grown in the presence of PCBs in solid media and their radial growth values were modelled through the Dantigny-logistic like function in order to estimate the time required by the fungal colonies to attain half their maximum diameter. The principal component analysis (PCA) revealed an inverse correlation between strain tolerance to PCBs and the laccase secretion over time, being laccase production closely associated with fungal growth capacity. Finally, a PCA was run to regroup and split between resistant and sensitive fungi. Simultaneously, a function associated with a model predicting the tolerance to PCBs was developed. Some of the assayed isolates showed a promising capacity to be applied in PCB bioremediation.

**Abbreviations:** Polychlorinated biphenyls (PCBs), white-rot fungi (WRF)

## 1. Introduction

Polychlorinated biphenyls (PCBs) are a group of chlorinated organic pollutants that have been used in a wide range of products in the twentieth century. Due to the harmful effect of PCBs on natural resources, their manufacture, use and export were banned but PCBs persist in the environment mainly because natural soil and aquatic biota are incapable of significantly degrading them (Borja et al. 2005). Although there are costly physical and chemical options for degradation of PCBs, fungal and bacterial bioremediation proved to be an effective and low-cost strategy for the removal of these pollutants. Previous studies not only showed that white-rot fungi (WRF) have the capability to degrade PCBs in liquid, soil and sediments but also demonstrated their adaptive ability to grow under critical environmental conditions (Stella et al. 2017). WRF such as *Phanerochaete chrysosporium* (Kamei et al. 2006a; Čvančarová et al. 2012), *Phlebia brevispora* (Kamei et al. 2006b), *Coriolus versicolor* (Cloete and Celliers 1999; Čvančarová et al. 2012), *Irpex lacteus, Bjerkandera adusta, Pycnoporus cinnabarinus, Phanerochaete magnolia, Pleurotus ostreatus* (Stella et al. 2017) and *Dichomitus squalen*s (Čvančarová et al. 2012) have already proved their potential for removal of PCBs. WRF possess a ligninolytic enzyme complex comprising, among other enzymes, lignin peroxidase (LiP, EC 1.11.1.14), manganese peroxidase (MnP, EC 1.11.1.13) and laccase (Lac, EC 1.10.3.2), involving as well in the oxidation of a wide range of organopollutants. Diverse studies shown that Lac, MnP and LiP were responsible for efficient degradation of PCBs (Č et al. 1997; Cloete and Celliers 1999; Gayosso-Canales et al. 2012). Other authors documented that these enzymes were involved in PCB bioremediation without establishing nevertheless their clear role in the removal mechanism (Pointing, 2001; Beaudette et al. 1998; Čvančarová et al. 2012).

Nevertheless, exploring new isolates with better degrading capacities would contribute in the field of bioremediation research. Different screening techniques were carried out to select strains with polycyclic aromatic hydrocarbons, dyes and phenols biodegradation capabilities (Levin et al. 2004; Matsubara et al. 2006; Lee et al. 2014; Carabajal et al. 2016). However, plate screening has not been yet evaluated as a quick technique to select strains capable of tolerating and degrading PCBs.

Predictive mycology has emerged as a new trend, dedicated to predict and understand fungal development in different raw materials and food (Dantigny et al. 2005; Dantigny and Bensoussan 2008; Bevilacqua et al. 2016).

The aim of the present study was to select a group of fungi capable of growing in the presence of PCBs, applying predictive mycology as a tool to assess their ability to tolerate PCBs. The tolerance of 26 white-rot fungal strains towards PCBs was compared in agar plates supplemented with PCBs by measuring radial growth rates and was correlated with their ligninolytic enzyme production.

## 2. Materials and methods

### 2.1. Organism and chemical compounds

Twenty-six white-rot fungal strains previously isolated mostly from the subtropical rainforest of Misiones (Argentina) were used in this work (Table 1) (González et al. 2013; Martínez et al. 2013). These strains are deposited at the Culture Collection of the Faculty of Forestry, Universidad Nacional de Misiones, Argentina, and maintained in malt extract (12.7 g L^−1^) and agar (17.0 g L^−1^) at 4°C.

**Table 1.** Fungal species studied.

Rn	Fungal species	Strain	Locality	Collection
1	*Phlebia subserialis*	C61	Campo Ramón, Misiones, Argentina	LBM 019
2	*Coriolus versicolor var. antarticus*	BAFC 266	Lago Filo Hua Hum, Neuquén, Argentina	BAFC 266
3	*Spongipellis pachyodon*	C24	Campo Ramón, Misiones, Argentina	LBM 012
4	*Ganoderma sp*.	JA7	Jardín América, Misiones, Argentina	LBM 001
5	*Irpex lacteus*	BAFC 1168D	Eldorado, Misiones, Argentina	BAFC 1168D
6	*Irpex lacteus*	BAFC 1171	Iguazú, Misiones, Argentina	BAFC 1171
7	*Lenzites elegans*	BAFC 2127	Santa Ana, Misiones, Argentina	BAFC 2127
8	*Ganoderma tornatum*	O4	Oberá, Misiones, Argentina	LBM 027
9	*Phanerochaete chrysosporium*	BKM-F 1767	USA	BKM-F 1767
10	*Pleurotus sajor-caju*	LBM 105	Eldorado, Misiones, Argentina	LBM 105
11	*Pycnoporus sanguineus*	A5	Posadas, Misiones, Argentina	LBM 020
12	*Pycnoporus sanguineus*	A7	Posadas, Misiones, Argentina	LBM 021
13	*Pycnoporus sanguineus*	C37	Campo Ramón, Misiones, Argentina	LBM 014
14	*Pycnoporus sanguineus*	KF1	Posadas, Misiones, Argentina	LBM 023
15	*Pycnoporus sanguineus*	L1	Posadas, Misiones, Argentina	LBM 024
16	*Pycnoporus sanguineus*	BAFC 2126	Garupá, Misiones, Argentina	BAFC 2126
17	*Pycnoporus* sp.	M1	Posadas, Misiones, Argentina	LBM 025
18	*Pycnoporus* sp.	A8	Posadas, Misiones, Argentina	LBM 022
19	*Pycnoporus* sp.	P21	Posadas, Misiones, Argentina	LBM 008
20	*Schizophyllum commune*	P12	Posadas, Misiones, Argentina	LBM 026
21	*Trametes elegans*	C53	Campo Ramón, Misiones, Argentina	LBM 017
22	*Trametes* sp.	C1	Campo Ramón, Misiones, Argentina	LBM 009
23	*Trametes villosa*	C2	Campo Ramón, Misiones, Argentina	LBM 010
24	*Trametes villosa*	C60	Campo Ramón, Misiones, Argentina	LBM 018
25	*Trametes villosa*	P4	Posadas, Misiones, Argentina	LBM 002
26	*Trametes villosa*	BAFC 2755	Posadas, Misiones, Argentina	BAFC 2755

The PCB mixture in transformed oil with an average of Aroclor 1242, 1254 and 1260 was kindly provided by KIOSHI S.A. (Buenos Aires, Argentina).

### 2.2. Tolerance for PCBs

Fungal strain tolerance towards PCBs was analysed in 90 mm diameter agar plates containing sugarcane bagasse-based medium [1 g (dry weight) sugarcane bagasse powder (120 mesh), 2 g KH_2_PO_4_, 0.5 g MgSO_4_·7H_2_O, 0.1 g CaCl_2_·2H_2_O, 0.1 mg FeSO_4_·7H_2_O and 10 g agar per litre distilled water (Matsubara et al. 2006)] supplemented with 85 mg L^−1^ of PCBs. Inocula consisted of a 5 mm diameter agar plug taken from a 5–7 day-old fungal colony in malt extract (12.7 g L^−1^) and agar (17.0 g L^−1^). Incubation was carried out at 28°C in darkness. Growth was followed by daily measuring the radial extension of the mycelium, until the complete coverage of the plates.

### 2.3. Ligninolytic enzyme assay

Ligninolytic activity was determined in solid media. All the strains were inoculated on agar plates (12 mL medium/Petri dish) containing malt extract (12.7 g L^−1^), glucose (10 g L^−1^) and agar (20 g L^−1^) (Malt Extract Agar with glucose (MEA)), supplemented either with 2,6-dimethoxyphenol (1 mM) (DMP) (Fonseca et al. 2010) to detect Lac activity, MnCl_2_·4H_2_O (1 mM) to estimate MnP (Jarosz-Wilkołazka et al. 2002) and Azure B (50 μM) to assay LiP (Archibald, 1992). Inocula consisted of a 5 mm colonised agar plug of a 5–7 day-old culture grown on MEA. Non-inoculated plates served as controls for abiotic discoloration. A MEA control plate was also inoculated with each strain. The plates were incubated at 28 ± 1°C, until the complete coverage of the plate. The Lac activity was confirmed when an orange–yellow staining zone appeared, while the MnP activity was observed as a brown staining zone. In the case of LiP activity, a decolorised zone appeared when the fungus degraded the dye. Three replicates were performed for each strain. Daily, the radial extension of the mycelium and the halos resulting from enzyme activity were measured.

### 2.4. Statistical analysis and modelling

Fungal growth was modelled by using a logistic equation, modified by Dantigny et al. (2011) and Bevilacqua et al. (2016) (Equation 1):

(1) $$D=\frac{D_{max}}{1+e^{k\left(\tau -t\right)}}$$

where *D* was the diameter of the fungal colony or the diameter of the halo resulting from enzyme activity, with *D*_max_ being the maximum diameter (set to 8.5 cm, corresponding to the diameter of the plates); *k* was the rate of fungal growth or the rate of enzyme activity on plate (cm day^−1^); τ was the time needed to attain half of *D*_max_ (days) and *t* was the time (days). Fitting was performed through the software InfoStat 2016p using a least squares approach with nonlinear regression (Di Rienzo et al. 2016).

Fungal growth τ was standardised as Δτ = τPCBs – τC, where τPCBs and τC were the values from medium supplemented with PCBs and control culture, respectively. A positive value of Δτ proved fungal growth inhibition in response to PCBs. Δτ,*k*Lac, *k*MnP, *k*LiP, τLac, τMnP and τLiP values were used as input values to run a principal component analysis (PCA) and joined the different isolates based on their tolerance. The correlation/probability matrix showed the interactive effect among the variables. A PCA was performed using Infostat 2016 software.

A validated cluster dendrogram was used to regroup fungi based on their tolerance for PCBs and the parameters obtained from the fit of the enzyme activity in solid media to the same model of fungal growth, and by means of nonlinear regression analysis Δτ were correlated as a function of the significant statistic variables.

In order to test growth response at different PCB concentrations, a group of selected fungi with different tolerances for PCBs was evaluated with increasing concentrations of PCBs and their Δτ were determined.

## 3. Results and discussion

Tolerance is one of the properties evaluated when searching microorganisms for bioremediation processes (Bayman and Radkar 1997; Matsubara et al. 2006; Lee et al. 2014; Sharma et al. 2017). A PCB tolerance test should be a primary stage in order to identify desirable PCB-degrading fungal species for biotechnological procedures. The results obtained in this work endorse predictive mycology as a tool and preliminary step to improve the screening and selection of strains capable of tolerating PCBs.

**Figure 1.**
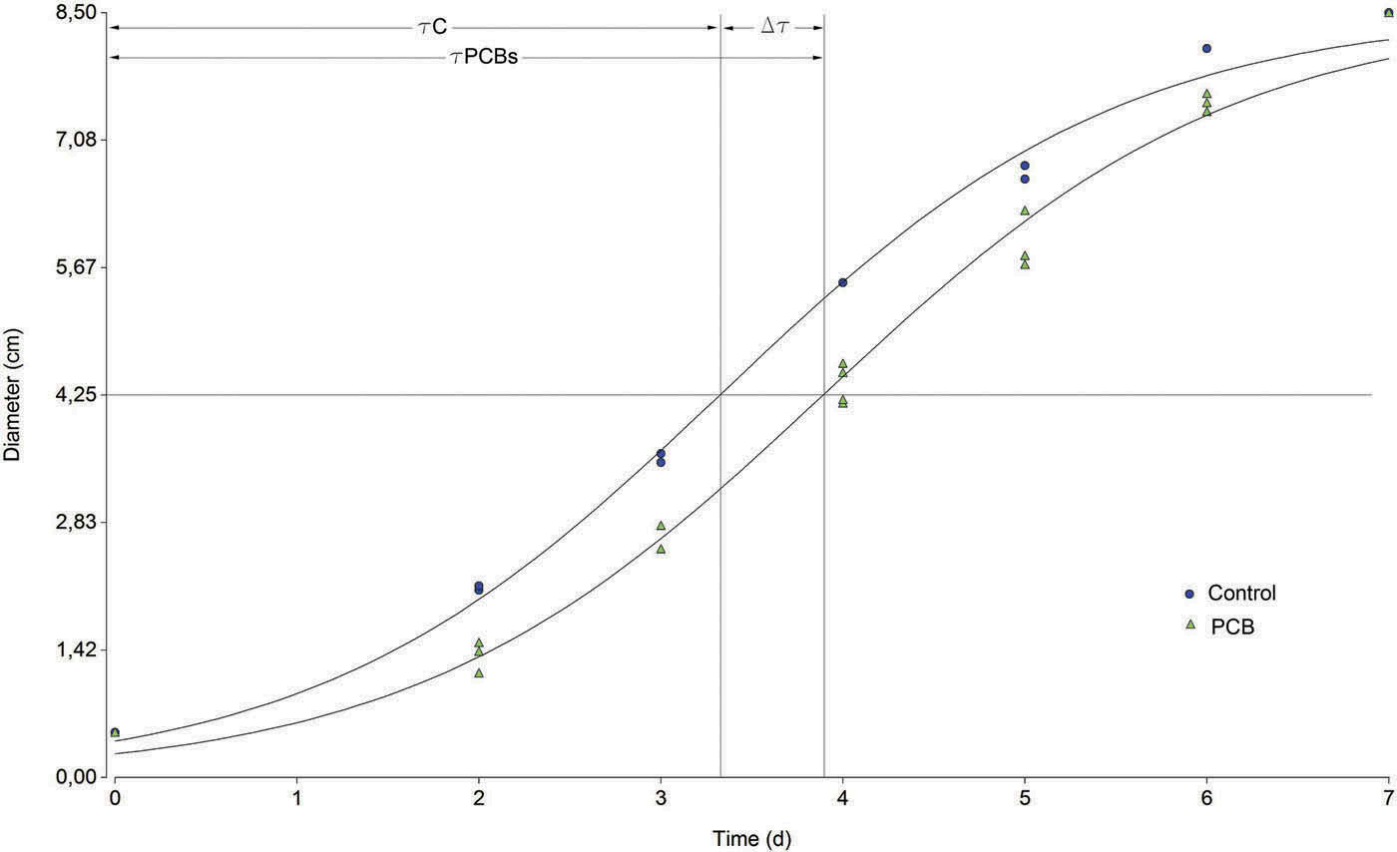
Example of diameter vs time plot for *Pleurotus sajor-caju* LBM 105 growing in both control and PCB-supplemented media.

The study of fungal growth kinetics on a solid culture medium supplemented with PCBs was conducted with Dantigny’s modified logistic model, fitting the equation to model the increase in diameter of the fungal colonies on plates. The modified equation of Bevilacqua et al. (2016) was used to fit the increase in diameter and it is intended to estimate the rate of fungal growth and the parameter τ, which could be used to compare the inhibition grade among isolates. Figure 1 shows an example of the diameter vs time plot of *Pleurotus sajor-caju* LBM 105 growing in both control and PCB-supplemented medium, and the fitting corresponding to Equation 1.

**Figure 2.**
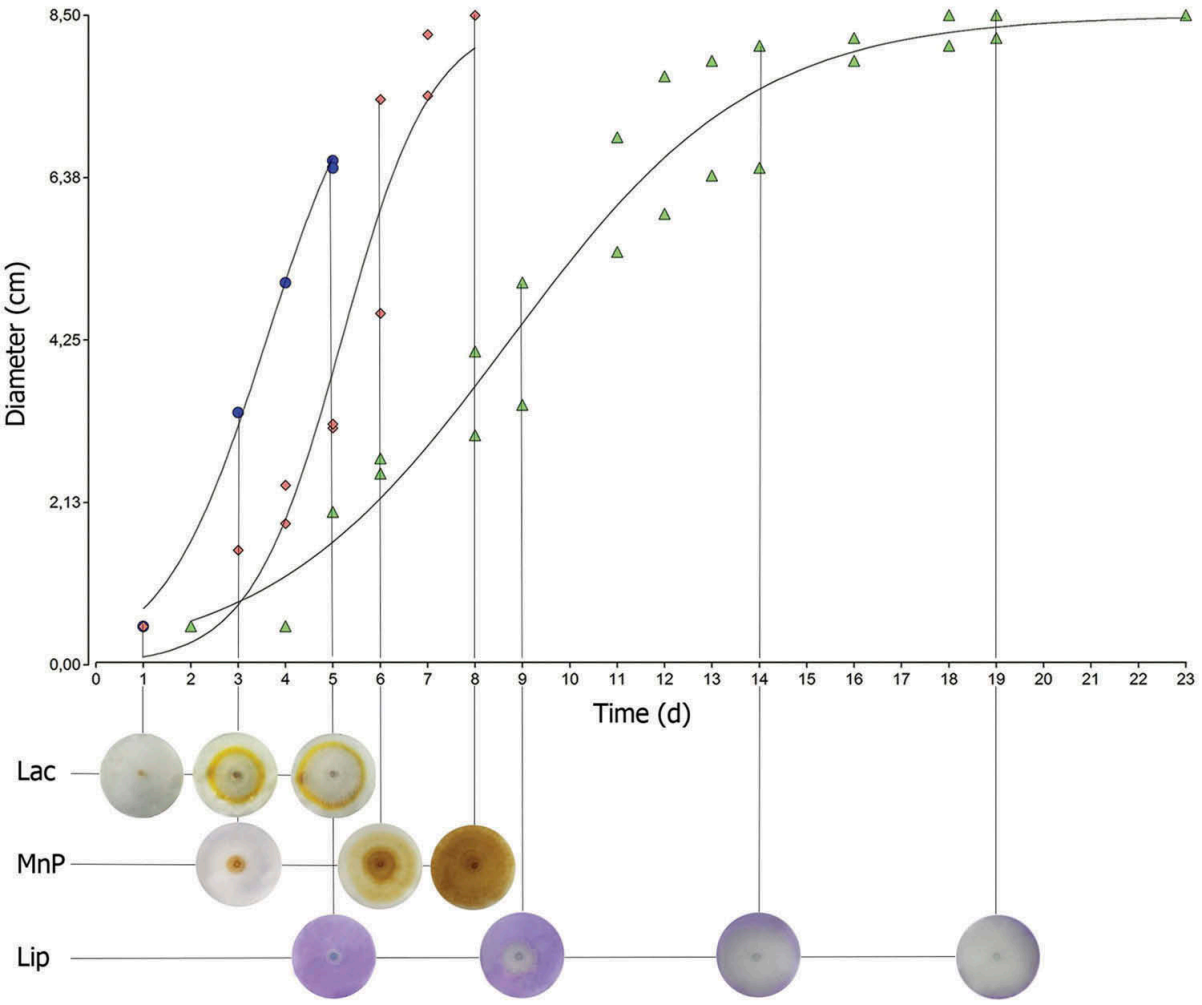
Nonlinear regression plot applied to *Pleurotus sajor-caju* LBM 105. Lac, MnP and LiP enzyme secretion adjusted to Bevilacqua’s equation.

Taking into account that the profiles of fungal enzyme activity on Petri dishes resembled those obtained for fungal growth, we adjusted the secretion halo of each enzyme to the same model and obtained *k* and τ as parameters of a quantitative and standardised characterisation. Figure 2 shows an example of the fitting in *Pleurotus sajor-caju* LBM 105 measurements of enzymes activity, and Table 2 presents Δτ, *k*Lac, τLac, *k*MnP, τMnP, *k*LiP and τLiP values obtained from the analysis of all isolates.

**Table 2.** Fungal τ (time to attain a half of the maximum diameter) (days) and *k* of ligninolytic activities (time/diameter of growth) on solid media.

Rn	Δτ	*k*Lac	τLac	*k*MnP	τMnP	*k*LiP	τLiP
1	0.26 ± 0.04	1.00 ± 0.01	2.93 ± 0.07	0.14 ± 0.02	11.19 ± 1.09	–	–
2	0.65 ± 0.02	0.94 ± 0.01	1.96 ± 0.03	0.81 ± 0.04	7.38 ± 0.04	0.67 ± 0.04	6.95 ± 0.05
3	1.22 ± 0.30	0.34 ± 0.01	3.56 ± 0.13	0.34 ± 0.02	9.24 ± 0.16	1.11 ± 0.04	9.82 ± 0.04
4	4.12 ± 0.01	0.33 ± 0.03	5.58 ± 0.36	-	-	-	-
5	0.31 ± 0.01	2.33 ± 0.29	3.72 ± 0.01	0.56 ± 0.02	6.71 ± 0.22	0.79 ± 0.01	5.30 ± 0.04
6	0.34 ± 0.01	0.69 ± 0.04	3.04 ± 0.08	0.23 ± 0.01	11.1 ± 1.08	-	-
7	0.36 ± 0.02	1.23 ± 0.01	3.17 ± 0.01	6.58 ± 0.24	4.1 ± 0.21	0.56 ± 0.01	5.92 ± 0.11
8	2.49 ± 0.04	0.56 ± 0.01	4.11 ± 0.11	-	-	-	-
9	0.93 ± 0.06	0.65 ± 0.01	4.84 ± 0.08	-	-	2.25 ± 0.12	4.28 ± 0.02
10	0.56 ± 0.01	0.99 ± 0.01	3.63 ± 0.01	1.10 ± 0.21	5.22 ± 0.33	0.43 ± 0.11	8.90 ± 1.17
11	1.44 ± 0.01	1.22 ± 0.07	3.10 ± 0.09	-	-	0.59 ± 0.03	5.31 ± 0.07
12	1.03 ± 0.01	1.00 ± 0.04	1.68 ± 0.04	0.44 ± 0.03	12.5 ± 0.59	0.74 ± 0.03	5.09 ± 0.19
13	1.00 ± 0.10	0.89 ± 0.02	2.71 ± 0.02	0.35 ± 0.01	14.65 ± 0.85	0.74 ± 0.02	4.29 ± 0.06
14	1.05 ± 0.03	1.13 ± 0.11	3.34 ± 0.04	-	-	0.54 ± 0.01	4.08 ± 0.04
15	0.96 ± 0.08	0.90 ± 0.03	1.83 ± 0.01	-	-	0.69 ± 0.01	4.38 ± 0.13
16	0.74 ± 0.01	0.78 ± 0.06	3.28 ± 0.07	-	-	0.48 ± 0.01	5.12 ± 0.02
17	1.34 ± 0.29	1.08 ± 0.01	2.29 ± 0.01	11.14 ± 0.06	11.99 ± 0.01	-	-
18	1.31 ± 0.06	1.12 ± 0.03	3.18 ± 0.07	1.17 ± 0.03	6.79 ± 0.05	0.55 ± 0.05	5.99 ± 0.16
19	1.01 ± 0.04	0.89 ± 0.10	2.83 ± 0.26	0.22 ± 0.01	15.51 ± 0.10	-	-
20	0.45 ± 0.12	0.48 ± 0.03	4.36 ± 0.01	-	-	-	-
21	2.98 ± 0.01	0.53 ± 0.01	4.98 ± 0.18	0.45 ± 0.01	13.37 ± 0.21	0.27 ± 0.04	10.43 ± 0.21
22	3.24 ± 0.01	0.35 ± 0.01	5.46 ± 0.14	-	-	0.33 ± 0.03	17.14 ± 0.32
23	1.02 ± 0.14	1.16 ± 0.03	2.32 ± 0.01	0.46 ± 0.01	5.55 ± 0.05	0.79 ± 0.09	5.08 ± 0.05
24	1.31 ± 0.06	1.00 ± 0.05	2.38 ± 0.02	3.19 ± 0.23	2.83 ± 0.01	0.50 ± 0.01	5.75 ± 0.09
25	1.24 ± 0.04	1.02 ± 0.04	2.53 ± 0.03	0.55 ± 0.49	6.17 ± 0.13	1.25 ± 0.01	4.49 ± 0.13
26	1.55 ± 0.01	1.36 ± 0.04	2.98 ± 0.04	0.83 ± 0.01	4.16 ± 0.12	0.87 ± 0.01	3.43 ± 0.04

*P. chrysosporium* was used as a reference fungus because it has been extensively applied as a model organism to study the physiological requirements and enzymes required for lignin and toxic biodegradation (Paszczynski and Crawford 1995).

**Figure 3.**
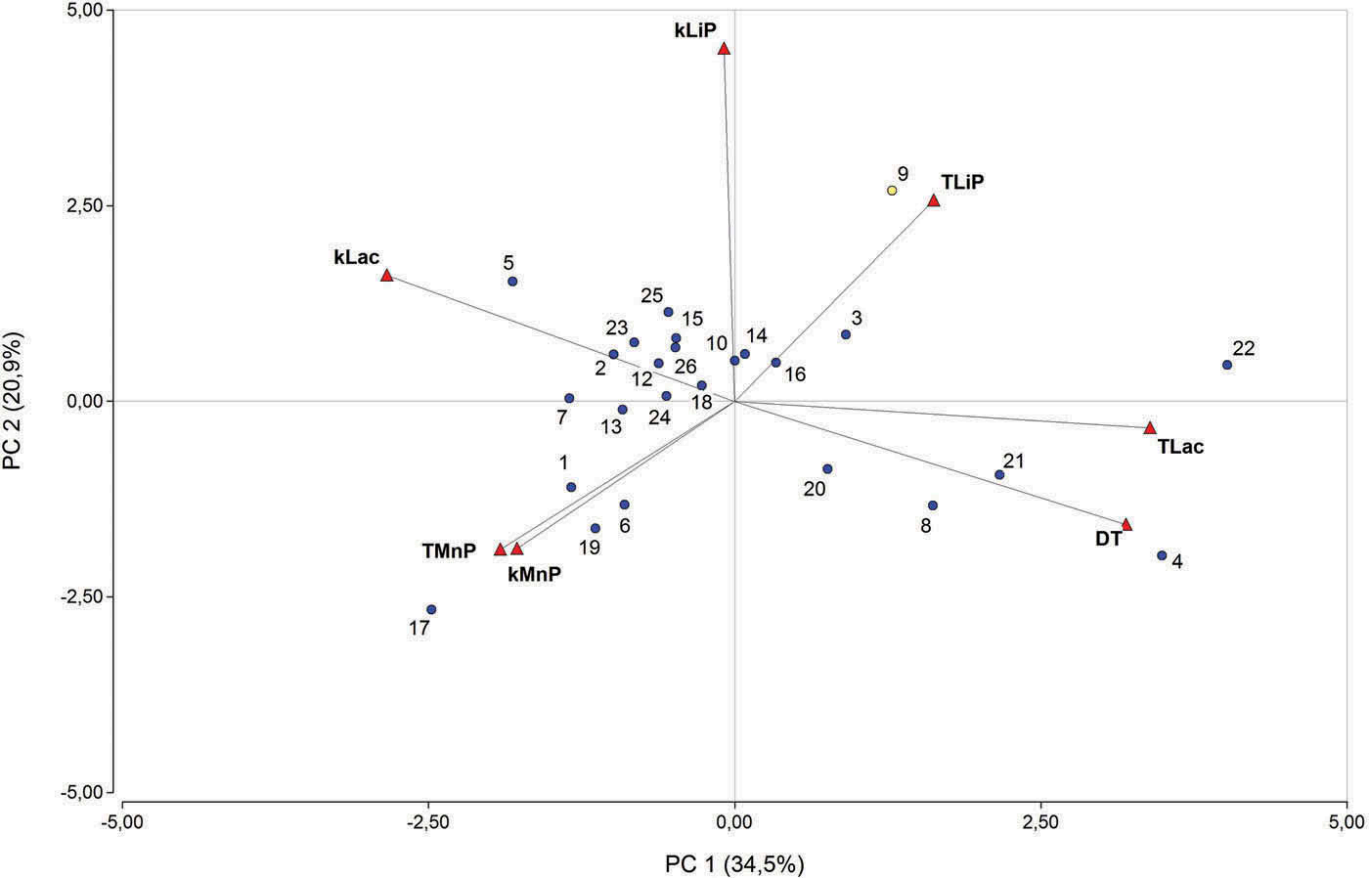
Score plot of PC1 vs PC2 illustrating the distribution of WRF, plotting with all variables. Fungal identification numbers are displayed in Table 1.

Figure 3 shows a biplot of the PCA with the fungal dispersion and the relative correlation among variables. The first two principal components (Supplementary Data, Table S1) with all variables explained 55.4% of the total variance. *k*Lac, τLac and Δτ were the main variables that described the variability among the fungi under study, and *k*LiP, *k*MnP and τMnP were the second main variables that explicated the variability among fungi (Supplementary Data, Table S2). Graphically, it can be seen as a high orthogonality between the MnP activity and the standardised inhibition Δτ (Supplementary Data, Table S3). The correlation/probabilities matrix verified that there was no probability of statistically significant correlation between MnP activity and Δτ (Supplementary Data, Table S4). Hence, it was possible to group the strains according to their tolerance. Forty-two percent of the fungi showed Δτ < 1, meaning that the difference between their growth rate in control and PCB-supplemented plates was minor or equal to 24 h. *P. chrysosporium* BKM-F 1767 belonged to this group. The other 42% of the fungi displayed a Δτ between 1 and 2, indicating that these strains grew 24 to 48 h slower in the presence of PCBs than in the control plates without pollutant. The rest of the strains (15%) revealed Δτ between 2 and 5, representing the group of fungi more severely affected by the presence of PCBs. *Ganoderma* sp. LBM 001 (Rn 4) showed the highest Δτ; therefore, because of its high sensibility it can be postulated as an indicator of toxicity in matrix contaminated with PCBs.

**Figure 4.**
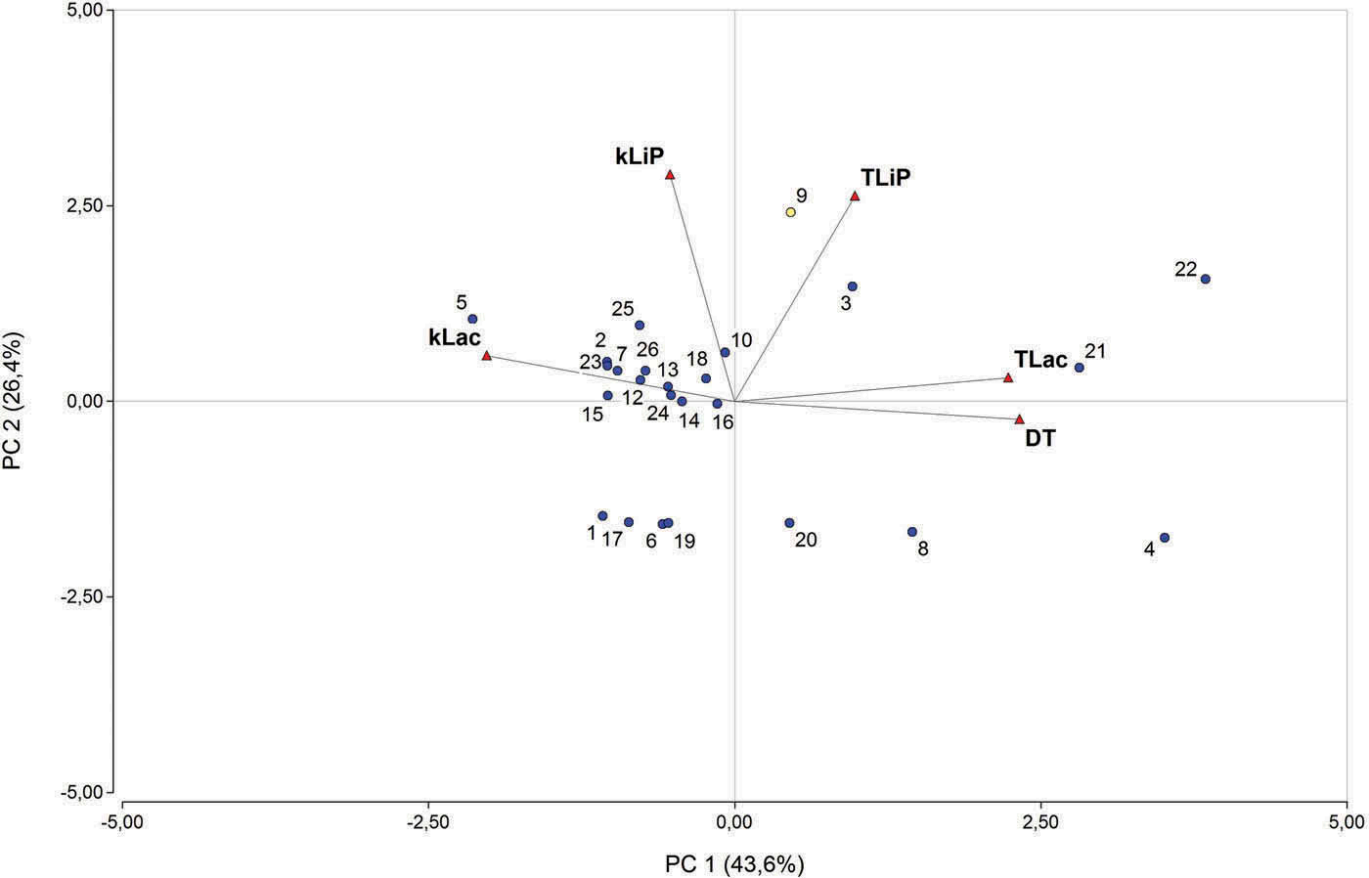
Score plot of PC1 vs PC2 illustrating the distribution of WRF, plotting after the elimination of *k*MnP and *τ*MnP. Fungal identification numbers are displayed in Table 1.

Removing the MnP activity in a second PCA (Figure 4), as analysis variable, total variability among observations was increased to 70%.

The Lac activity and Δτ proved to be the main variables that explained variability among observations (Supplementary Data, Table S5), with a strong negative correlation between *k*Lac and Δτ. This was verified by means of the correlation/coefficients matrix (Supplementary Data, Table S6) between Δτ and *k*Lac of −0.50 (*p*-value < .05), and Δτ and τLac of 0.62 (*p* < .05) (Supplementary Data, Table S7). The second PCA also showed that *Trametes* sp. LBM 009, *Trametes elegans* LBM 017 and *Ganoderma* sp. LBM 001 (Rn 22, Rn 21 and Rn 4) were mostly differentiated by this effect in comparison with isolates of *Phlebia subserialis* LBM 019, *C. versicolor var. antarticus* BAFC 266, *I. lacteus* BAFC 1168 D, *Pycnoporus* sp. LBM 025 and *Trametes villosa* LBM 010 (Rn1, Rn 2, Rn 5, Rn 17 and Rn 23). The LiP activity resulted in the second main cause of variability among strains, with *Spongipellis pachyodon* LBM 012, *I. lacteus* BAFC 1168D, *P. chrysosporium* BKM-F 1767, *Trametes* sp. LBM 009 and *T. villosa* LBM 002 (Rn 3, Rn 5, Rn 9, Rn 22 and Rn 25) being mainly differentiated from *Phlebia subserialis* LBM 019, *Ganoderma* sp. LBM 001, *I. lacteus* BAFC 1171, *Ganoderma tornatum* LBM 027, *Pycnoporus* sp. LBM 025, *Pycnoporus* sp. LBM 008 and *Schizophyllum commune* LBM 026 (Rn 1, Rn 4, Rn 6, Rn 8, Rn 17, Rn 19 and Rn 20). Graphically, an elevated orthogonality between the variable Δτ and the LiP activity was observed, implying independence between these variables.

Although this is a novel assay for tolerance for PCBs in solid media, the obtained results are in agreement with previous studies which demonstrated a noteworthy role of Lac in the presence of this pollutant. In addition, MnP and LiP had a secondary effect on fungal tolerance. Several authors have established a relationship between Lac from WRF and their capability for degradation of PCBs (Dietrich et al. 1995; Novotný et al. 1997; Moeder et al. 2005; Plačková et al. 2012). Gayosso-Canales et al. (2012) proved that among the ligninolytic enzymes versatile peroxidase, MnP and Lac, only the Lac activity was implicated in the removal of highly chlorinated PCBs. Placková et al. (2012) demonstrated an increase in Lac secretion in the presence of PCBs without establishing a clear connection with the degradation of this pollutant. They suggested that in addition to the role of Lacs in biodegradation, these enzymes might display other functions in fungal cultures. Lac secretion has been connected with pathogenicity, protecting fungal pathogens from toxic phytoalexins and tannins in the host environment (Bollag et al. 1988; Pezet et al. 1991). The same detoxification function might explain the higher tolerance for PCBs of the fungi tested, that secreted Lac in a shorter period in comparison with those with a slower secretion rate. Among previous studies, a correlation between the ability of various WRF to produce ligninolytic enzymes and their capability of removing PCBs in liquid media was reported by Novotny et al. (1997; 2004); nevertheless, Krčmář et al. (1999) and Chroma et al. (2002) could not found a correlation between MnP and LiP activities and fungal ability to degrade PCBs.

**Figure 5.**
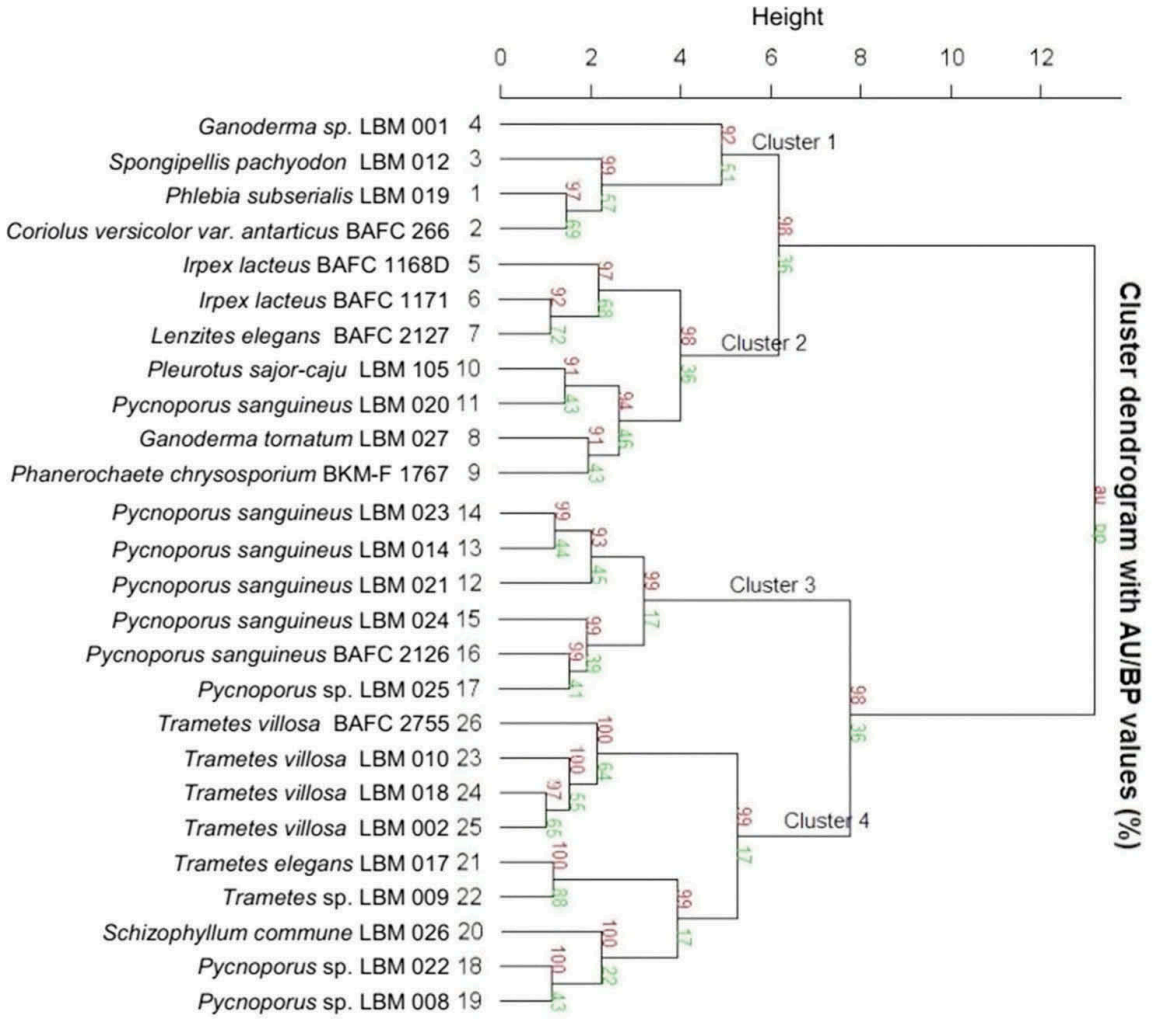
Validated dendrogram of 26 fungal isolates based on their Δτ, Lac activity and LiP activity, prepared using Infostat 2016 software linked to R software.

The results of 25 fungal isolates and the control strain resulting when assaying tolerance for PCBs were joined in a single cluster analysis in order to produce a dendrogram derived from InfoStat software combined with pvcluster package from R software to facilitate their comparison (Figure 5). Pvclust calculates probability values (*p*-values) for each cluster using bootstrap resampling techniques. Two types of *p*-values are available: approximately unbiased *p*-value and bootstrap probability value (Suzuki and Shimodaira 2006).

Through a dendrogram cluster, the probability of clusters with an average method and Euclidean distance was verified, evidenced by four main clusters of 98%, 92%, 99% and 99% probability of similarity respectively among strains. The divisive coefficient was high (0.891), indicated a good clustering structure. The clustering tree provided four clusters based on the ability of the fungal isolates to tolerate PCBs and their Lac and LiP activities. Cluster 1 consisted of four isolates without specific and common characteristics. Cluster 2 consisted of seven isolates with a high capacity to tolerate PCBs and high Lac activity. Cluster 3 consisted of six isolates with an intermediate tolerance for PCBs and intermediate Lac activity, with a particularly presence of fungi of *Pycnoporus* genus. Finally, cluster 4 consisted of nine isolates characterised by low tolerance for PCBs with low Lac and LiP activities, which can be useful strains in order to test toxicity.

In order to identify the best-fit model between Δτ and Lac activity, the Pearson correlation coefficients between Δτ and τLac elevated at different exponents were compared (Supplementary Data, Table S8). Considering the correlation coefficient 0.88 of Δτ^2^ and τLac^3^, we proceeded to adjust Δτ^2^ with the simple variable τLac by means of the following nonlinear function:

(2) $$\Delta \tau^{2}\;=\;A\;\times \;\tau Lac\;+\;B\;\times \;\tau Lac^{2}\;+\;C\;\times \;\tau Lac^{3}$$

The resulting fitting model equation was (Supplementary Data, Table S9):

(3) $$\Delta \tau^{2}\;=3.9522\tau Lac-2.3840\tau Lac^{2}\;+\;0.3844\tau Lac^{3}$$

**Figure 6.**
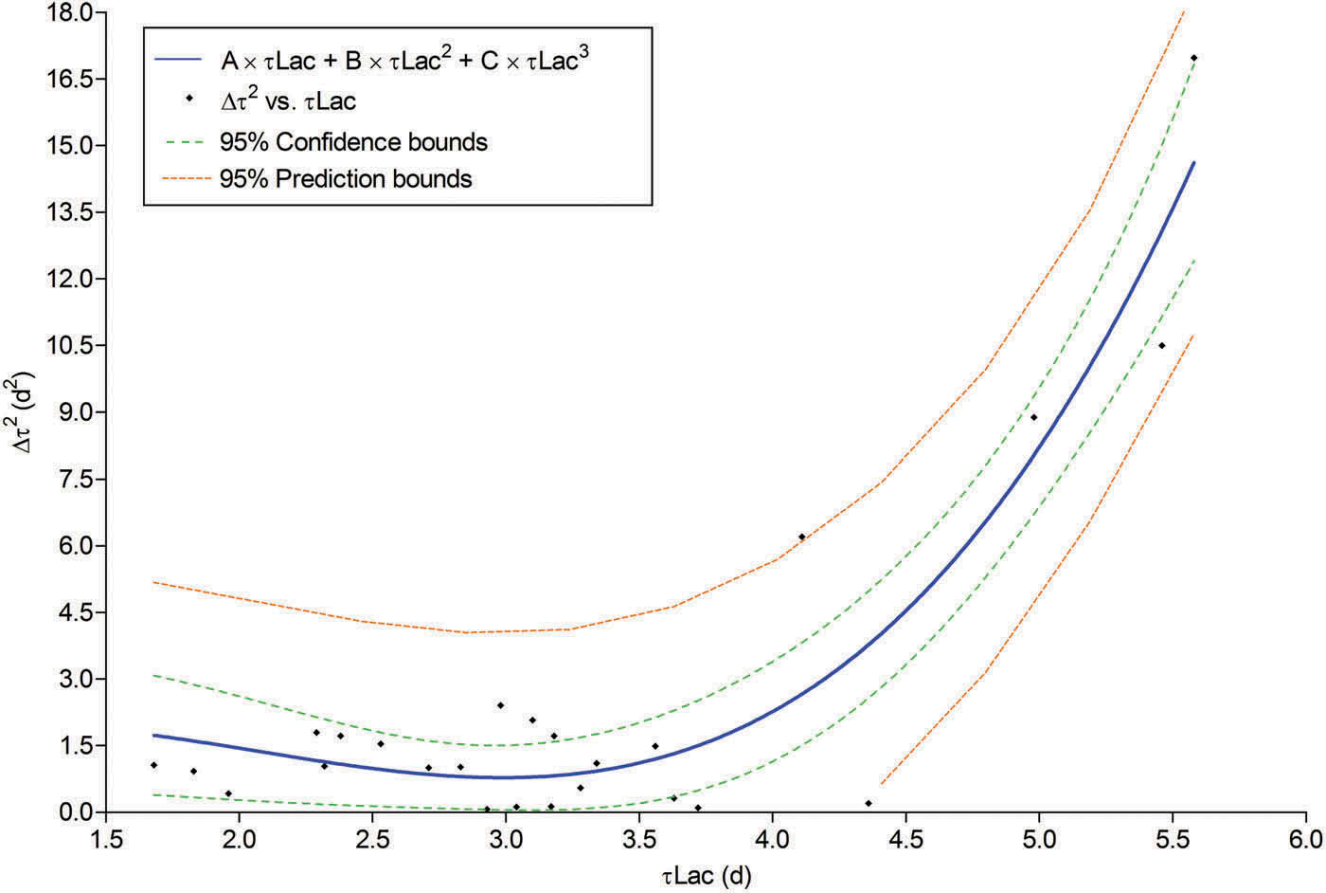
Function and fitted model of Δτ^2^ with the simple variable τLac.

The *R*^2^-statistic = 86.73% explains the variability in Δτ^2^ and the adjusted *R*^2^-statistic was 85.53%. The Durbin–Watson statistic (DW = 2.44041) proved the absence of significant correlation based on the order in which the data were presented (Figure 6). Although it is well known that *R*^2^-statistic, in itself, cannot be used to decide whether a new model is an improvement (Baranyi and Roberts 1995), the fitted mathematical function presented in this paper corroborates the previously obtained PCA and dendrogram results.

A leave-one-out cross-validation method (Supplementary Data, Data S10) was used to obtain coefficients (Supplementary Data, Table S11) that were compared as independent samples. At *p* > .05, the null hypotheses *H*(0) = *H*(0′) was not rejected; therefore, the model equation was validated. Equation 3 was validated in the range of Δτ [0.26; 4.12] and τLac [1.68; 5.58].

The obtained function expressed that low-slung τLac values (high Lac secretion) predict low Δτ values (degree of inhibition) towards PCB presence, and therefore high PCB fungal tolerance.

It was developed a general function to predict the tolerance for PCBs of different basidiomycetous fungi based on their Lac activity, and might be applied to select fungal strains suitable for bioremediation processes. 

**Figure 7.**
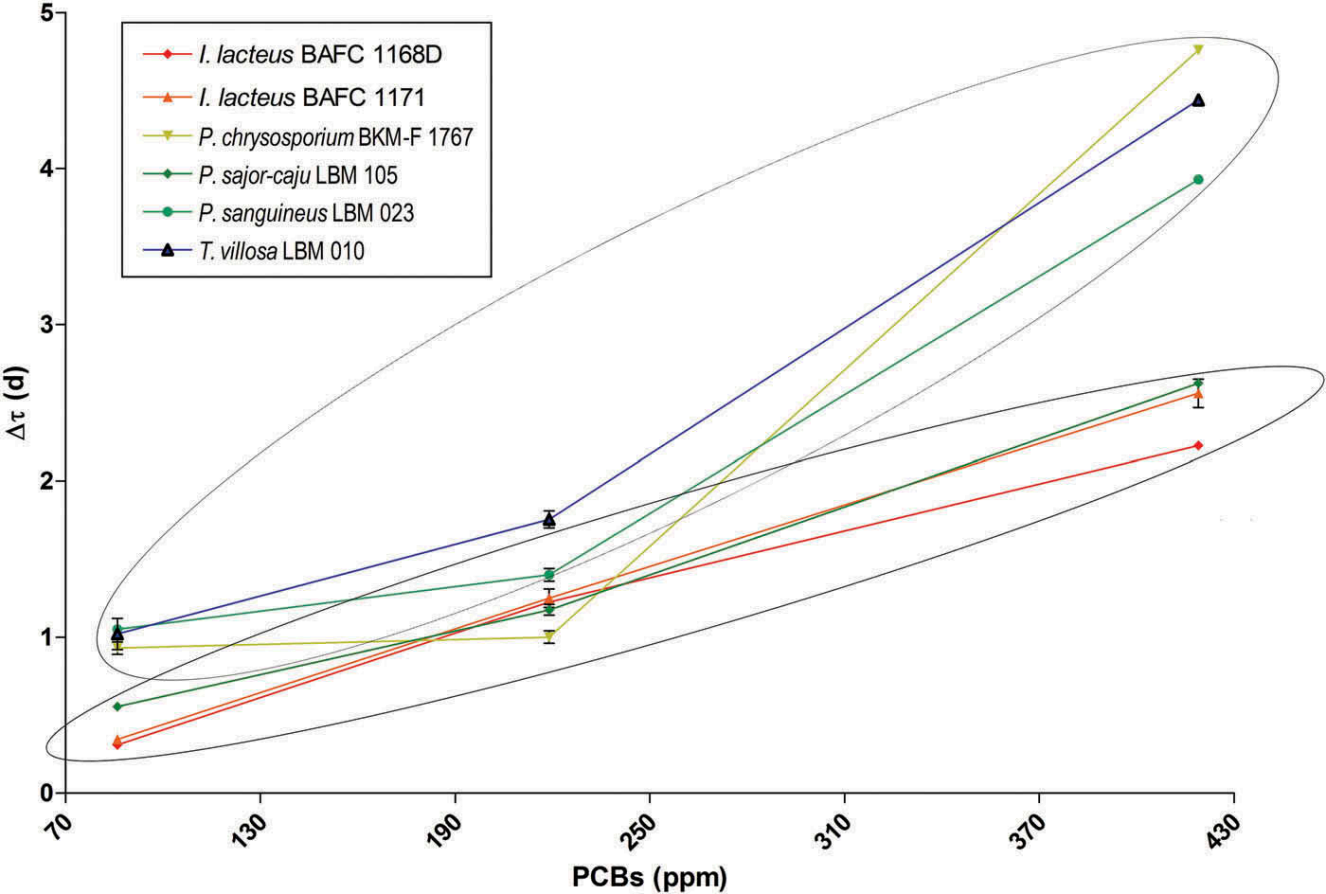
Δτ determination at three different PCB concentrations and identification of two homogenates fungal groups with *p* < .05.

In order to evaluate the effect of the increase in PCB concentration on fungal growth, the rate of mycelial growth in agar plates was measured in six selected fungi based on their high, intermediate and low capacity to tolerate PCBs (Figure 5). Δτ slightly augmented with increasing pollutant concentrations, and two levels of tolerance appeared, depicted in Figure 7 (Δτ vs PCB concentration). An Analysis of Variance (ANOVA) determined a significant statistical difference with *p* < .05 between two fungal groups. *I. lacteus* BAFC 1171, *I. lacteus* BAFC 1168D and *P. sajor-caju* LBM 105 exhibited the lowest Δτ for various PCB concentrations. The other group significantly different was composed by *P. chrysosporium* BKM-F 1767, *P. sanguineus* LBM 023 and *T. villosa* LBM 010 which showed minor tolerance for PCBs. Figure 7 shows that the tolerance ability of each fungus at low levels to PCBs remains similar at high levels of PCBs.

The observed decrease in fungal growth while increasing PCB concentration is in agreement with previous studies (Murado et al. 1976). This negative effect of PCBs on fungal growth rate allows assuming that tolerance for PCBs is a reliable indicator of fungal ability to co-metabolise these pollutants (Yin et al. 2011).

## 4. Conclusions

This is the first screening evaluating growth and ligninolytic enzyme production by WRF in a solid medium amended with PCBs, as a strategy to select tolerant fungi to these pollutants. It opens the possibility of identifying more strains that might be suitable as PCB bioremediation agents, linking the use of predictive microbial growth as a part of a low time-consuming and health-security model, complemented with conventional microbiological selection techniques.

The principal component analysis (PCA) revealed a strong correlation between the degree of inhibition and the activity of the enzyme Lac (*p* < .05), and to a lesser extent with non-significant statistical difference (*p* > .05) with the activity of the enzyme LiP. The MnP activity did not show influence on the degree of inhibition (*p* > .05).

Effective bioremediation requires the selection of species with desirable characteristics such as high tolerance and enzymatic activity; thereby, from the obtained results, *I. lacteus* BAFC 1168D, *I. lacteus* BAFC 1171, *L. elegans* BAFC 1227, *P. sajor-caju* LBM 105 could be ranked as promising PCB degraders. Among these four strains, *P. sanguineus* LBM 014, *P. sanguineus* LBM 021 and *P. sanguineus* LBM 023 had also good competitive activity, based on the observed tolerance model associated with Lac activity. *I. lacteus* BAFC 1171, *I. lacteus* BAFC 1168D and *P. sajor-caju* LBM 105 exhibited the lowest Δτ for different PCB concentrations, indicating a relative proportional tolerance for an increase in PCB concentration.

The next step will be to test the best strains for their potential to remediate PCB-contaminated substrates, also considering their possible use as a fungal consortium.

Based on the mean PCA and the cluster analysis, three isolates from cluster 2 were selected for a second-stage screening on a rice-straw-powder-amended medium. *I. lacteus* BAFC 1171, *I. lacteus* BAFC 1168D and *P. sajor-caju* LBM 105, which displayed the highest tolerance for PCBs, might be valuable as a useful resource for the biodegradation of PCBs and a variety of other biotechnological applications.

Finally, an applied equation was developed that can be used to predict the tolerance for PCBs of WRF based on their Lac activity in solid media supplemented with PCBs, and might be applied to select fungal strains suitable for their bioremediation.

## Geolocation information

27°26′11.4″S 55°53′14.9″W
