# Supplementary material for: Assessing the ability of white-rot fungi to tolerate polychlorinated biphenyls using predictive mycology
Source: Mycology. 2018 Jun 8;9(4):239–49. doi: 10.1080/21501203.2018.1481152 (PMC6282474; doi:10.1080/21501203.2018.1481152)
Supplement: Supplemental Material [file TMYC_A_1481152_SM7318.docx]

**Supplementary Table S1.** ACP1 Eigenvalues

| λ | Value | Proportion | Cumulative proportion |
| --- | --- | --- | --- |
| **1** | **2.42** | **0.35** | **0.35** |
| **2** | **1.46** | **0.21** | **0.55** |
| 3 | 0.95 | 0.14 | 0.69 |
| 4 | 0.82 | 0.12 | 0.81 |
| 5 | 0.55 | 0.08 | 0.88 |
| 6 | 0.47 | 0.07 | 0.95 |
| 7 | 0.34 | 0.05 | 1.00 |

**Supplementary Table S2.** ACP 1 Eigenvectors

| Variables | e1 | e2 |
| --- | --- | --- |
| Δτ | 0.51 | -0.25 |
| *k*Lac | -0.45 | 0.26 |
| τLac | 0.54 | -0.05 |
| *k*MnP | -0.28 | -0.30 |
| τMnP | -0.31 | -0.30 |
| *k*LiP | -0.01 | 0.72 |
| τLiP | 0.26 | 0.41 |

**Supplementary Table S3.** ACP1 Correlation/Coefficients matrix.

|  | Δτ | *k*Lac | τLac | *k*MnP | τMnP | *k*LiP | τLiP |
| --- | --- | --- | --- | --- | --- | --- | --- |
| Δτ | 1.00 |  |  |  |  |  |  |
| *k*Lac | -0.50 | 1.00 |  |  |  |  |  |
| τLac | 0.62 | -0.46 | 1.00 |  |  |  |  |
| *k*MnP | -0.11 | 0.22 | -0.28 | 1.00 |  |  |  |
| τMnP | -0.21 | 0.14 | -0.33 | 0.21 | 1.00 |  |  |
| *k*LiP | -0.20 | 0.17 | -0.05 | -0.17 | -0.19 | 1.00 |  |
| τLiP | 0.24 | -0.10 | 0.25 | -0.13 | -0.11 | 0.30 | 1.00 |

**Supplementary Table S4.** ACP1 Correlation/Probabilities matrix.

|  | Δτ | *k*Lac | τLac | *k*MnP | τMnP | *k*LiP | τLiP |
| --- | --- | --- | --- | --- | --- | --- | --- |

| Δτ |  |  |  |  |  |  |  |
| --- | --- | --- | --- | --- | --- | --- | --- |
| *k*Lac | 0.0100 |  |  |  |  |  |  |
| τLac | 0.0010 | 0.0211 |  |  |  |  |  |
| *k*MnP | 0.5989 | 0.2910 | 0.1757 |  |  |  |  |
| τMnP | 0.3185 | 0.4895 | 0.1114 | 0.3146 |  |  |  |
| *k*LiP | 0.3283 | 0.4273 | 0.8105 | 0.4161 | 0.3505 |  |  |
| τLiP | 0.2498 | 0.6214 | 0.2318 | 0.5458 | 0.6153 | 0.1388 |  |

**Supplementary Table S5.** ACP2 Eigenvectors

| Variables | e1 | e2 |
| --- | --- | --- |
| Δτ | 0.59 | -0.06 |
| *k*Lac | -0.51 | 0.15 |
| τLac | 0.56 | 0.08 |
| *k*LiP | -0.13 | 0.73 |
| τLiP | 0.25 | 0.66 |

**Supplementary Table S6.** ACP2 Correlation/Coefficients matrix.

|  | Δτ | *k*Lac | τLac | *k*LiP | τLiP |
| --- | --- | --- | --- | --- | --- |
| Δτ | 1.00 |  |  |  |  |
| *k*Lac | -0.50 | 1.00 |  |  |  |
| τLac | 0.62 | -0.46 | 1.00 |  |  |
| *k*LiP | -0.20 | 0.17 | -0.05 | 1.00 |  |
| τLiP | 0.24 | -0.10 | 0.25 | 0.30 | 1.00 |

**Supplementary Table S7.** ACP2 Correlation/ Probabilities matrix.

|  | Δτ | *k*Lac | τLac | *k*LiP | τLiP |
| --- | --- | --- | --- | --- | --- |
| Δτ |  |  |  |  |  |
| *k*Lac | 0.0100 |  |  |  |  |
| τLac | 0.0010 | 0.0211 |  |  |  |
| *k*LiP | 0.3283 | 0.4273 | 0.8105 |  |  |
| τLiP | 0.2498 | 0.6214 | 0.2318 | 0.1388 |  |

**Supplementary Table S8.** Pearson correlation coefficients between Δτ and τLac elevated at different exponents.

| Variable(1) | Variable(2) | n | Pearson correlation coefficient | *p*-value |
| --- | --- | --- | --- | --- |
| Δτ | τLac | 25 | 0.66 | <0.05 |
| Δτ | τLac ^2^ | 25 | 0.74 | <0.05 |
| Δτ | τLac ^3^ | 25 | 0.79 | <0.05 |
| Δτ ^2^ | τLac | 25 | 0.75 | <0.05 |
| Δτ ^2^ | τLac ^2^ | 25 | 0.82 | <0.05 |
| **Δτ ^2^** | **τLac ^3^** | **25** | **0.88** | **<0.05** |
| Δτ ^3^ | τLac | 25 | 0.74 | <0.05 |
| Δτ ^3^ | τLac ^2^ | 25 | 0.82 | <0.05 |
| Δτ ^3^ | τLac ^3^ | 25 | 0.87 | <0.05 |

**Supplementary Table S9.** Nonlinear regression results

|  |  |  | Intervalo Confianza a | 95,00% |
| --- | --- | --- | --- | --- |
|  |  | Error Estándar | Asintótico | |
| Parámetro | Estimado | Asintótico | Inferior | Superior |
| a | 3.95217 | 1.22512 | 1.41143 | 6.49291 |
| b | -2.38396 | 0.64617 | -3.72403 | -1.04388 |
| c | 0.384399 | 0.0801669 | 0.218143 | 0.550655 |

**Supplementary data S10.** Validation command used in MATLab R2008b.

clear all; clc;

τLac=[2.93 1.96 3.56 5.58 3.72 3.04 3.17 4.11 3.63 3.1 1.68 2.71 3.34 1.83 3.28 2.29 3.18 2.83 4.36 4.98 5.46 2.32 2.38 2.53 2.98];

Δτ =[0.26 0.65 1.22 4.12 0.31 0.34 0.36 2.49 0.56 1.44 1.03 1 1.05 0.96 0.74 1.34 1.31 1.01 0.45 2.98 3.24 1.02 1.31 1.24 1.55];

Δτ 2=Δτ .^2;

x=τLac; y=Δτ 2;

N=length(x);

sse=0;

for i = 1:25

[train,test] = crossvalind('LeaveMOut',N,1);Intercept =0;

F = @(B,x) B(3).*x.^3+B(2).*x.^2+B(1).*x+Intercept;

beta0 = [1 1 1 0];

Beta = nlinfit(x(train), y(train), F, beta0);J=[Beta(1) Beta(2) Beta(3) 0];GG(i,1)=Beta(1);GG(i,2)=Beta(2);GG(i,3)=Beta(3);

yhat = polyval(J,x(test));

sse = sse + sum(((yhat - y(test))/(1-cov(y(test))/var(y(train)))).^2);sseM(i)=sse;

end

CVerr = sse / 25

GG

**Supplementary Table S11.** Validation coefficients obtained by "Cross Validation"

| a | b | c |
| --- | --- | --- |
| 3.9385 | -2.3788 | 0.3839 |
| 4.1995 | -2.5059 | 0.3986 |
| 3.9245 | -2.3594 | 0.3808 |
| 4.0564 | -2.4329 | 0.3886 |
| 3.9398 | -2.3806 | 0.3842 |
| 3.9245 | -2.3594 | 0.3808 |
| 3.9398 | -2.3806 | 0.3842 |
| 3.8111 | -2.3183 | 0.3771 |
| 3.9498 | -2.3730 | 0.3824 |
| 3.9724 | -2.3843 | 0.3837 |
| 3.9788 | -2.4165 | 0.3896 |
| 3.9731 | -2.3937 | 0.3855 |
| 3.9673 | -2.3944 | 0.3858 |
| 3.7934 | -2.2870 | 0.3719 |
| 3.7934 | -2.2870 | 0.3719 |
| 4.2212 | -2.5180 | 0.4001 |
| 3.8111 | -2.3183 | 0.3771 |
| 3.7934 | -2.2870 | 0.3719 |
| 4.0564 | -2.4329 | 0.3886 |
| 3.7934 | -2.2870 | 0.3719 |
| 3.8111 | -2.3183 | 0.3771 |
| 3.9932 | -2.4095 | 0.3877 |
| 3.9731 | -2.3937 | 0.3855 |
| 3.7035 | -2.2350 | 0.3654 |
| 3.9385 | -2.3788 | 0.3839 |
